# The effect of rod orientation on electrical anisotropy in silver nanowire networks for ultra-transparent electrodes

**DOI:** 10.1038/srep34289

**Published:** 2016-09-28

**Authors:** Thomas Ackermann, Raphael Neuhaus, Siegmar Roth

**Affiliations:** 1 Graduate School of Excellence in Advanced Manufacturing Engineering, University of Stuttgart, Nobelstr. 12, 70569 Stuttgart, Germany; 2 Fraunhofer Institute for Manufacturing Engineering and Automation, Nobelstr. 12, 70569 Stuttgart, Germany; 3 Institute for Industrial Manufacturing and Management, University of Stuttgart, Allmandring 35, 70569 Stuttgart, Germany; 4 Alan G. MacDiarmid NanoTech Institute, University of Texas at Dallas, 800 West Campbell Rd., Richardson, TX 75080, USA; 5 Sineurop Nanotech GmbH, Muenchner Freiheit 6, 80802 Munich, Germany

## Abstract

Two-dimensional networks made of metal nanowires are excellent paradigms for the experimental observation of electrical percolation caused by continuous jackstraw-like physical pathways. Such systems became very interesting as alternative material in transparent electrodes, which are fundamental components in display devices. This work presents the experimental characterization of low-haze and ultra-transparent electrodes based on silver nanowires. The films are created by dip-coating, a feasible and scalable liquid film coating technique. We have found dominant alignment of the silver nanowires in withdrawal direction. The impact of this structural anisotropy on electrical anisotropy becomes more pronounced for low area coverage. The rod alignment does not influence the technical usability of the films as significant electrical anisotropy occurs only at optical transmission higher than 99 %. For films with lower transmission, electrical anisotropy becomes negligible. In addition to the experimental work, we have carried out computational studies in order to explain our findings further and compare them to our experiments and previous literature. This paper presents the first experimental observation of electrical anisotropy in two-dimensional silver nanowire networks close at the percolation threshold.

Alternative materials for transparent conductive films (transparent electrodes) in displays and touch panels have sparked huge interest during the past years^1^,^2^,^3^. The use of the conventional material indium tin oxide (ITO) demands high process temperatures and expensive indium mining. Moreover, it cannot be used within a bendable device due to its brittleness. Frequently discussed alternative materials are graphene^4^,^5^, carbon nanotube (CNT) networks^6^,^7^ and networks of metallic nanowires^8^,^9^,^10^, especially silver nanowires (AgNWs). With regard to optoelectrical performance, AgNWs are superior to the other alternative materials^11^. Industrial requirements for transparent electrodes are often referred as sheet resistance *R*_*s*_ < 100 Ω/sq and optical transmission T > 90%^12^,^13^,^14^. Multiple works have shown that such performance is achieved easily^9^,^15^,^16^. However, AgNW films appear hazy, which does not match today’s quality standards in display industry. Haze increases with area coverage and wire diameter. We have previously shown that only networks made of rather thin AgNWs (diameter d = 31 nm) with area coverage that corresponds to T > 97% exhibit acceptable low haze^11^,^17^. For d = 25 nm we observe the same requirements (Fig. S2, supporting information). In case of such ultra-transparent films, sufficient conductivity (*R*_*s*_ < 100 Ω/sq) can only be achieved by using AgNWs with very high aspect ratio and amongst hundreds of papers on transparent AgNW films, only three works achieve this requirement^11^,^18^,^19^.

AgNW films are usually prepared by liquid film coating. Among other liquid film coating techniques such as Meyer rod coating, knife coating and slot die coating, dip-coating is a self-metered coating technique and the liquid film is created by withdrawing the substrate from an ethanolic AgNW dispersion (Fig. 1a). After evaporation of the ethanol, the AgNWs create a continuous two-dimensional network that allows electrical conductivity. It is important to mention that structural percolation means that the number of AgNWs per area unit is high enough in order to create a continuous physical path along the entire film, whereas electrical percolation means continuous diffusion of electrons along the network. Certainly structural percolation is required for electrical percolation.

Figure 1b shows the shape of the meniscus for different withdrawal velocities *u*_*w*_. According to the Landau-Levich law, the liquid film thickness *h*_0_ depends on *u*_*w*_ as *h*_0_ ∝ 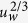 20. For *u*_*w*_ between 20 and 1000 mm/min, *h*_0_ is between 1 and 15 *μ*m. We found *u*_*w*_ = 300 mm/min to be a withdrawal velocity that creates a liquid film with a thickness that allows the ethanol to evaporate quickly enough for potential upscaling of the coating process^11^. The dimensions of the AgNWs used in this work are length L = 19 ± 9 *μ*m and diameter d = 25 ± 5 nm. Figure 1c shows the length histogram of the wires. It is clearly seen that L is significantly larger than *h*_0_ = 6.6 *μ*m in case of *u*_*w*_ = 300 mm/min. Thus, due to the restricted translational and rotational degree of freedom in z-direction, the withdrawn AgNWs may be aligned as we know it from chromosome combing in biophysics^21^. Recently such alignment of AgNWs caused by dip-coating was observed^11^,^22^. Furthermore the superiority of dip-coating over spray-coating with regard to resulting optoelectrical performance was shown. However, the alignment of AgNWs has to be discussed further for comprehensive understanding of anisotropic percolation.

The key issue of this paper is the examination of structural anisotropy within ultra-transparent AgNW networks and its impact on electrical anisotropy. Pioneer research on anisotropic stick percolation dates back to the 1980s^23^. Since systems of percolating rodlike nanostructures became technically very relevant during the past years, anisotropic stick percolation has sparked more interest again. Some works are solely based on simulations^24^,^25^, whereas others include experimental findings^26^,^27^,^28^. All of these studies discuss two-dimensional networks of aligned CNTs. These systems exhibit multiple experimental challenges and influencing parameters such as the amount of metallic CNTs, lattice defects, bundle size (since CNTs are often aggregated in smaller bundles), aspect ratio and the necessity of stabilizing surfactants in order to create CNT inks. Hence, CNT networks are complex and comparison of literature becomes difficult. Networks of AgNWs feature less influencing parameters and higher conductivity, which permits the observation of electrical percolation much closer to the percolation threshold. Therefore AgNW networks are better paradigms for the description of rod alignment and its impact on electrical percolation. Recently Jagota *et al*. have carried out simulations on such systems^29^, and here we show the first experimental work on electrical anisotropy of AgNW networks. We have produced about 150 AgNW films on glass substrates with optical transmission in the range of 97.5 to 99.8%. For multiple samples the sheet resistance of the films was measured in x- and in y- direction in order to investigate electrical anisotropy. Scanning force microscopy has been used for the investigation of the rod alignment. Moreover, we compare our experimental results with our own simulations and simulations from literature.

## Results

### Optoelectrical performance of the ultra-transparent AgNW films

Before we discuss anisotropy effects, we discuss the optoelectrical performance of the ultra-transparent AgNW films. The optical transmission T has been controlled by changing the colloid concentration. The initial Ag concentration was c(Ag) = 2.5 w% and stepwise dilution to 0.06 w% allows the production of AgNW films with different T. One dip results in optical transmission between 97.5 and 99.8%. In Fig. 2 we plot the optoelectrical performance T(*R*_*s*_). The sheet resistance *R*_*s*_ is measured between two silver paste contacts in x-direction (*R*_*s*,*x*_ in Fig. 3a). We note that four-probe measurements have been tested and have resulted in similar values as the two-probe measurements. Hence, the contact resistance between the silver contact and the AgNW film can be neglected for further discussion. It is clearly seen that the industrial requirement of *R*_*s*_ < 100 Ω/sq is achieved although T is rather high (vertical line in Fig. 2). Thus, the optoelectrical performance can be regarded as very good. In order to describe T(*R*_*s*_) in a quantitative way, we applied curve fitting to our data following De *et al*. who describe T(*R*_*s*_) with percolation theory^12^.


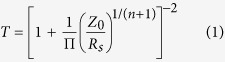


Equation 1 derives from the percolation scaling law 1/*R*_*s*_ ∝ (*N* − *N*_*c*_)^*n*^, where N is the number of conductive rods per area unit with *N*_*c*_ as the critical rod number required for percolation, and n is the percolation coefficient. The dimensionless figure of merit Π describes the optoelectrical quality of a two-dimension network made of percolating conductors, and *Z*_0_ is the impedance of free space (377 Ω). High values of Π and low values of n are desirable. Our curve fitting results in Π = 244 and n = 0.88. This figure of merit is significantly higher than previously reported^12^. Only two other works show similar optoelectrical performance^18^,^19^.

It is seen that Equation 1 does not properly describe the most transparent samples in Fig. 2. Recently we have reported that n is not constant within one system and increases towards lower area coverage^17^. Moreover, the percolation scaling law and hence Equation 1 is not valid for *N*/*N*_*c*_ < 1.3, according to recent Monte-Carlo simulations^30^. It is important to mention that the accuracy of the spectrometer is 0.1% which influences exact detection of the actual percolation threshold. However our experimental results show good agreement with theory. The optical transmission T, the area coverage A and the number of rods per area unit N correspond to the following relation.


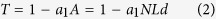


**Figure 1:**
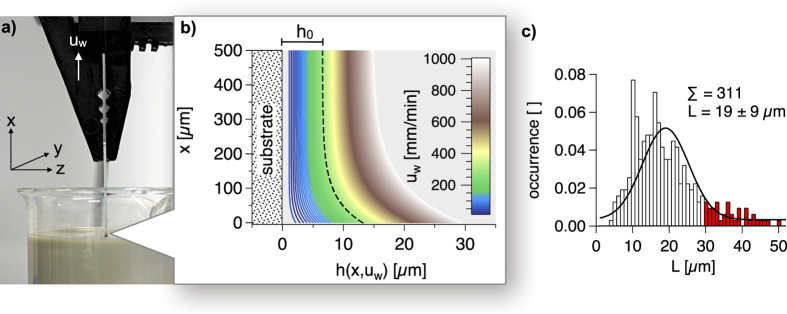
(**a**) A substrate withdrawn from an ethanolic AgNW dispersion. (**b**) Profiles of the meniscus for different withdrawal velocity *u*_*w*_. The dashed curve represents the shape for the withdrawal velocity applied in this work (300 mm/min) which results in a liquid film thickness of *h*_0_ = 6.6 *μ*m. A mathematical description of the meniscus can be found in the supporting information. (**c**) Length distribution of the AgNWs used in this work. The normal distribution has its mean at 19 *μ*m and the standard deviation is 9 *μ*m. The highlighted part of the histogram represents very long AgNWs with L > 30 *μ*m.

**Figure 2:**
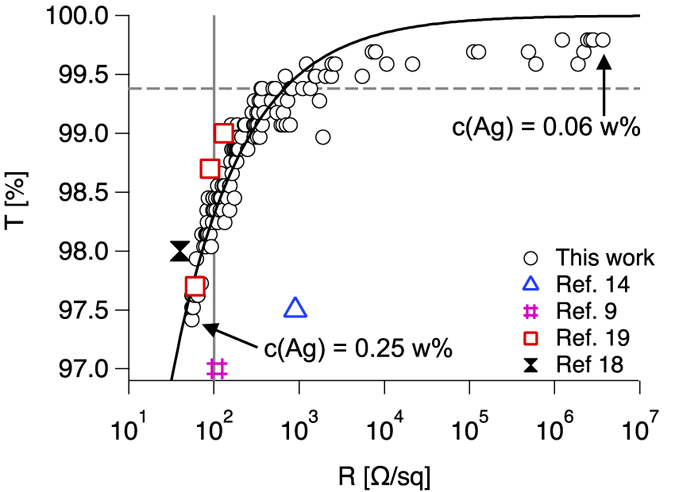
The optical transmission T versus the sheet resistance *R*_*s*_. The vertical line represents industrially required *R*_*s*_ < 100 Ω/sq. Curve fitting has been applied following Equation 1. The dashed line represents the percolation threshold expected from percolation theory.

The correction factor *a*_1_ considers light scattering and depends on the rod diameter d and the wavelength of the incidenting light. It has been found *a*_1_ = 0.87 for thin AgNWs and wavelength of 550 nm^17^,^31^. We know from percolation theory that the critical rod number per area unit *N*_*c*_ that is required for percolation scales with the rod length L as *N*_*c*_ = 5.637/*L*^2 ^32,33. For the AgNWs with L = 19 *μ*m, we expect from Equation 2 the percolation threshold to be located at T = 99.4% (Fig. 2, dashed line). The actual percolation threshold, where electrical conductivity is still detectable, at *T*_*p*_ = 99.8% implies that only a third of the theoretically required number of rods already cause electrical percolation. We attribute this deviance from theory to two different circumstances. The first one concerns the length distribution of the AgNWs shown in Fig. 1c. The highlighted fraction of AgNWs with L > 30 *μ*m is 12%. Simulations of Mutiso *et al*. have shown that only a small amount of very long AgNWs can significantly increase the optoelectrical performance^14^. The second circumstance that may lead to a shift of the actual percolation threshold is partial alignment of the AgNWs. Monte-Carlo simulations of Behnam *et al*. predict that two-dimensional networks of rodlike conductors exhibit their maximum of conductivity in case of partially aligned rods compared to random orientation^24^,^25^. Having said that, extensive alignment causes strong decrease of the conductivity for similar N. In the further discussion we will present a closer look at the alignment of the AgNWs and its impact on electrical percolation.

### Experimental observation of electrical and structural anisotropy

In order to observe films with low haze that is not detectable for the human eye, the optical transmission has to be higher than 97% (Fig. S2 in the supporting information). The results presented in Fig. 2 show that our low-haze and ultra-transparent films achieve the industrial requirement of *R*_*s*_ < 100 Ω/sq. However, *R*_*s*_ was measured between two silver paste contacts that allow only the determination of *R*_*s*_ in withdrawal direction of the dip-coating process (x-direction, *R*_*s*,*x*_). Touch panels have to exhibit conductivity in both dimensions of the display. Therefore the conductivity in y-direction (*R*_*s*,*y*_) should be in the same range.

**Figure 3:**
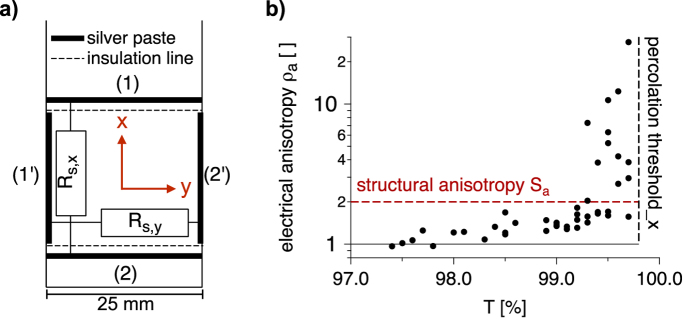
(**a**) Approach for the measurement of electrical anisotropy. (**b**) The macroscopic electrical anisotropy quotient *ρ*_*a*_ at different optical transmission. The vertical dashed line represents the experimental percolation threshold from Fig. 2 (T = 99.8%).

Multiple samples from Fig. 2 have been characterized with regard to *R*_*s*,*y*_. Figure 3a shows the sketch of the procedure. After measuring *R*_*s*,*x*_ between the silver lines (1) and (2), contact lines (1′) and (2′) are coated on the edges of the substrate and insolation lines are scratched with a peaked scalpel. Before measuring *R*_*s*,*y*_ between (1′) and (2′), we made sure that there are no shortcuts between neighbored silver lines (e.g. (1) and (1′)). In Fig. 3b we plot the ratio of the two sheet resistances of several samples versus their optical transmission, and define the macroscopic electrical anisotropy quotient *ρ*_*a*_:


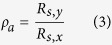


For samples with T > 99%, pronounced electrical anisotropy is observed. For samples with T < 98.5%, *R*_*s*,*y*_ is still larger than *R*_*s*,*x*_, but the electrical anisotropy is significantly less distinct (*ρ*_*a*_ < 1.3) than it is for lower area coverage, respectively higher T. As long as both *R*_*s*,*x*_ and *R*_*s*,*y*_ are <100 Ω/sq, such slight electrical anisotropy would not be a drawback for the use in touch panels. Moreover, *ρ*_*a*_ is ≈1 for T = 97.5%, where *R*_*s*_ is 55 Ω/sq. Hence, electrical anisotropy does not influence the industrially relevant samples. Only those samples with unacceptable conductivity (T > 99%) exhibit significant electrical anisotropy.

The occurrence of pronounced electrical anisotropy at low area coverage leads us straightly to the question how the orientation of the AgNWs influences the conductivity. Orientation of the AgNW may occur due to the dimension of the aforementioned Landau-Levich meniscus (Fig. 1a).

**Figure 4:**
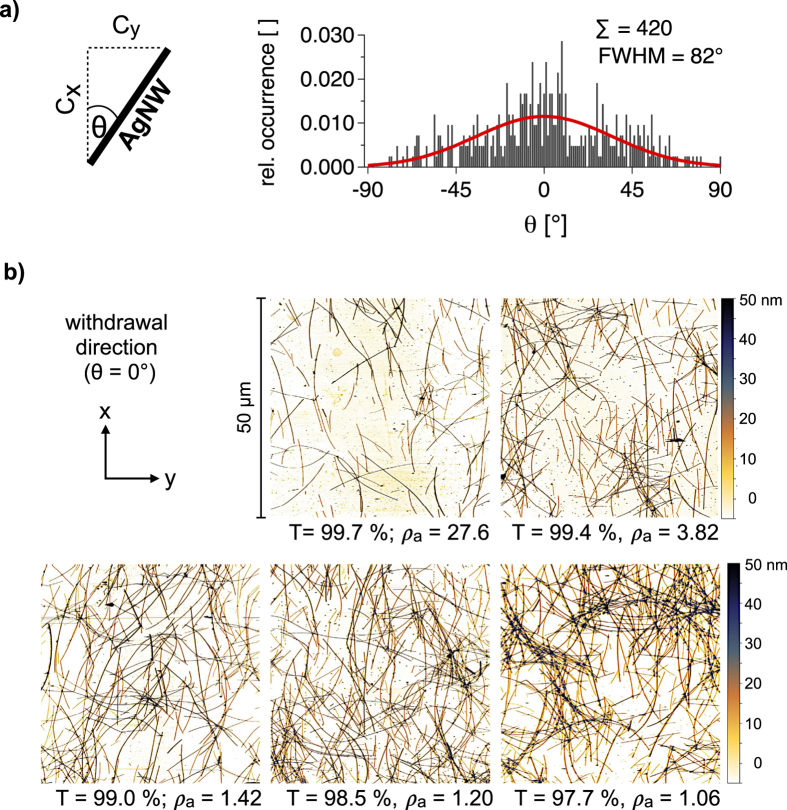
(**a**) Distribution of the orientation angle *θ* determined by scanning force microscopy. The sum of all counts is normalized to 1. (**b**) Scanning force micrographs of several AgNW films with different optical transmission T and macroscopic electrical anisotropy quotient *ρ*_*a*_.

In case of dominant rod orientation in x-direction, less AgNWs should be required for percolation in x-direction than for percolation in y-direction. Each rod is aligned on the xy-plane with a x- and a y-component (*C*_*x*_, *C*_*y*_). The scale of the two components is determined by the orientation angle *θ* (−90° ≤ *θ* ≤ 90°) as shown in Fig. 4a. Except for *θ* = ±45°, the rods have either a larger x- or y-component. In case of macroscopic structural anisotropy with the rod number per area unit N, either *θ* is other than −90° ≤ *θ* ≤ 90° or the distribution of *θ* is other than random. For an ensemble of sticks, the macroscopic orientation components are *C*_*x*_ = NL · cos *θ* and *C*_*y*_ = NL · sin *θ*. Analogously to the electrical anisotropy (Equation 3), the structural anisotropy can be described as follows, where *S*_*a*_ is the macroscopic structural anisotropy quotient.


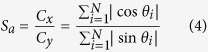


In order to describe structural anisotropy statistically, we have analyzed scanning force micrographs of AgNW networks with low area coverage (T > 99.5%), where the determination of *θ* is easier because the AgNWs are aligned more isolated than at higher area coverage. It is important to mention that we used the same withdawal velocity (300 mm/min) for all samples since this parameter likely influences the ordering of the wires. The first micrograph in Fig. 4b represents a scanning force micrograph of such an AgNW network with low area coverage. All micrographs in Fig. 4b clearly show dominant alignment towards the withdrawal direction (x) of the dip-coating process. We note that due to tip artifacts commonly observed in scanning force microscopy the wires appear thicker than they actually are. In Fig. S4 of the supporting information, we address this effect more detailed and show that the area coverage calculated from the micrographs is in good agreement with the area coverage calculated from the optical transmission T after applying a correction factor.

We have measured *θ* for 420 AgNWs. Multiple AgNWs have slight curvature, especially longer AgNWs. Assuming continuous curvature, we have determined *θ* from the line between the AgNW ends, which corresponds to the tangent at the middle of the AgNWs. In Fig. 4a we show the distribution of *θ*. The fraction of AgNWs with more distinct *C*_*x*_ corresponding to −45° < *θ* < 45° is 78% in the histogram and 81% in the Gaussian distribution. This result is clearly different from isotropic structural percolation (50%). The full width at half maximum (FWHM) of the Gaussian distribution is FWHM = 82°. Applying Equation 4 to the data of the histogram results in *S*_*a*_ = 1.99. Conceivably the distribution of *θ* may be different for higher area coverage. Moreover it is more difficult to analyse such samples as mentioned earlier. However the supporting information provides a histogram similar to Fig. 4a for T between 98.1 and 99.0% (Fig. S6) and we observe FWHM = 78° instead of 82° and *S*_*a*_ = 2.14 instead of 1.99. Hence, *S*_*a*_ does not appear to be entirely independent from the area coverage. The reason why the predominant orientation of the AgNWs slightly changes for denser samples is because the AgNWs might not have as much space to rotate in the liquid film before encountering another wire, compared to samples with lower area coverage. However, we consider the simplification of *S*_*a*_ being ≈2 for all samples is sufficiently accurate for the further discussion.

We can see that *S*_*a*_ is larger than *ρ*_*a*_ for AgNW films with T < 99% (Fig. 3b). For area coverage close to the percolation threshold, *ρ*_*a*_ is significantly larger than *S*_*a*_. The micrographs in Fig. 4b indicate that there are more AgNWs contributing to percolation in withdrawal direction (x) than in y-direction in case of low area coverage (T = 99.7%). Increasing the number of AgNWs leads to less distinct electrical anisotropy until the network reaches electrical isotropy (T = 97.7, *ρ*_*a*_ ≈ 1). Moreover, we would expect higher *N*_*c*_ for detectable conductivity in y-direction. Indeed we found a slight difference of the percolation threshold between the two directions (*T*_*p*_(*x*) = 99.8%, *T*_*p*_(*y*) = 99.7%). *T*_*p*_ is the highest observed transmission with detectable conductivity. However the instrumental accuracy for T is 0.1%. For a closer examination of the critical rod number per area unit *N*_*c*_ and its relation to the rod orientation *θ*, we have carried out computational studies.

### Computational approach and comparison to experimental data

**Figure 5:**
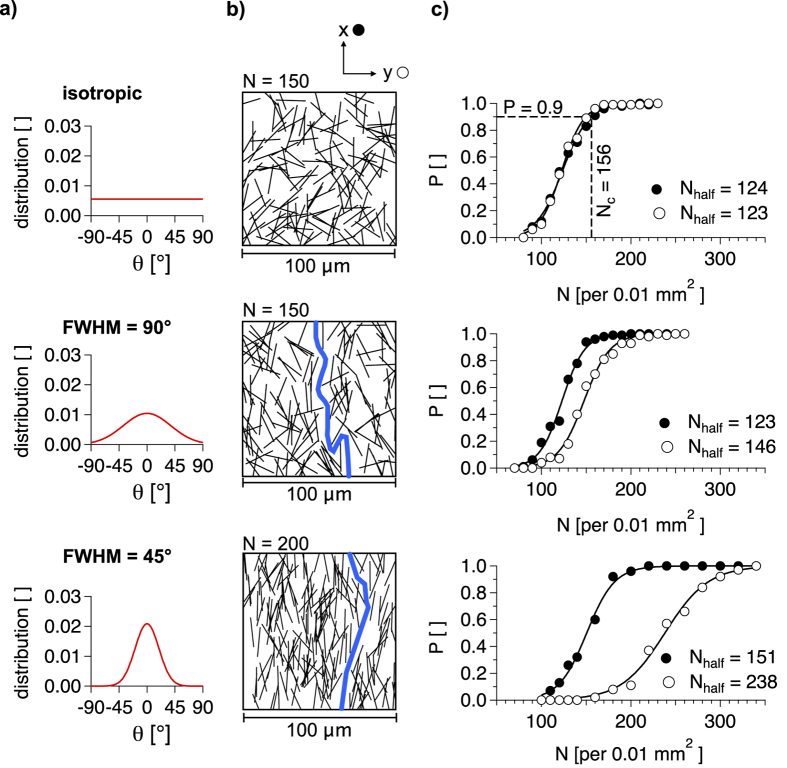
(**a**) The angular distribution of the orientation angle *θ* for different extent of vertical alignment. The area under the curves is normalized to 1. (**b**) Simulated AgNW networks for the three angular distributions, where N is the number of sticks per 0.01 mm^2^. The thick paths represent insulating gaps which prohibit percolation in y-direction. (**c**) The percolation probability P versus the rod number N. Full markers represent the percolation probability in x-direction (*P*_*x*_) and empty markers in y-direction (*P*_*y*_). The curves correspond to fitting a sigmoid function to the data (Equation 5).

To obtain a network of randomly positioned wires, we have seeded N points within a square representing an edge dimension of 100 *μ*m in Mathematica^®^. The wires are generated by defining the randomly seeded points as centres between the two stickends. The distance between the stickends equals the length of the wires (19 *μ*m). The supporting information provides detailed information about the computational procedure. The orientation *θ* of the sticks is generated randomly in case of isotropic percolation and by a Gaussian distribution in case of anisotropic percolation. Figure 5a represents the angular distributions that have been applied to the simulations. The total occurrence equals 1. The angular Gaussian distribution *G*_*θ*_(*μ*, *σ*) is described by its centre at the mean value *μ* = 0° and the standard deviation *σ*, which can be expressed by the FWHM as FWHM = 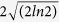 *σ*. Here we apply a random angular distribution and two Gaussian angular distributions with FWHM = 90° and 45° (Fig. 5a). We have varied the number of wires per area unit N for each angular distribution. For each N the random network generation has been applied a hundred times and it is examined whether or not a percolation path is obtained in x- and y- direction. Figure 5b shows examples for generated networks and in Fig. 5c we plot the percolation probabilities *P*_*x*_ and *P*_*y*_ versus N for the three angular distributions. As expected, we observe *P*_*x*_(*N*) = *P*_*y*_(*N*) in case of isotropic percolation. It is clearly seen that predominant alignment of the wires leads to separation of *P*_*x*_(*N*) and *P*_*y*_(*N*) for lower N. Multiple generated networks exhibit continuous percolation paths in x-direction but there are insulating gaps in y-direction, represented by the thick lines in Fig. 5b. The separation of *P*_*x*_(*N*) and *P*_*y*_(*N*) becomes more distinct for lower FWHM (45°). At N = 200, *P*_*x*_ is almost 1, whereas *P*_*y*_ is 0.1. The data can be described with a sigmoid function, which is expressed as follows.


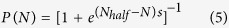


The key parameters of Equation 5 are the centre of the curve (N at P = 0.5, *N*_*half*_) and the steepness s. For most of the curves in Fig. 5c s is between 0.07 and 0.08, except for *P*_*y*_(*N*) at FWHM = 45°, where s is 0.04. Hence, the intermediate state where percolation sets on becomes broader for percolation in y-direction if the alignment of the sticks increases. As mentioned before, we know from percolation theory that the critical rod number per area unit *N*_*c*_ that is required for percolation scales with the rod length L as *N*_*c*_ = 5.637/*L*^2 ^32,33. However, this scale applies only for isotropic orientation. For L = 19 *μ*m we calculate *N*_*c*_ = 156 per 0.01 *mm*^2^. Equation 5 results in P(156) = 0.9 for isotropic percolation (Fig. 5c). There are several possibilities for the definition of the actual percolation threshold. On the one hand, we can define it as the N, where P(N) becomes 1 and on the other hand it can be defined as the inflection point of the sigmoid function (*N*_*half*_), where P is 0.5^34^. Other works have reported the percolation threshold for bond percolation to be at P = 0.77^35^,^36^. Regardless of the actual definition of the percolation threshold we observe a separation of *P*_*x*_(*N*) and *P*_*y*_(*N*), especially for angular distribution with FWHM = 45°.

**Figure 6:**
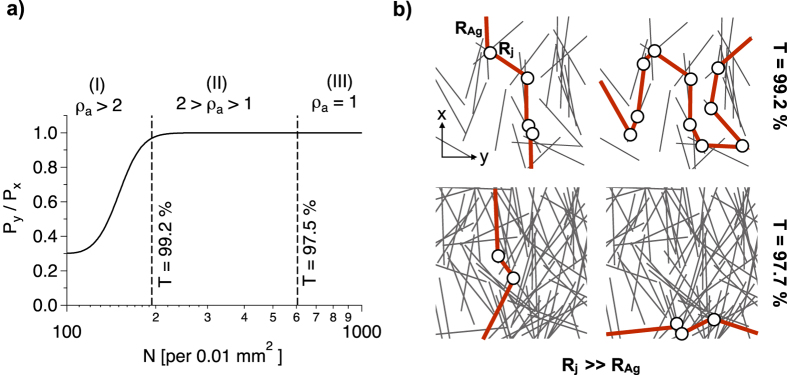
(**a**) The ratio of the percolation probabilies in y- and x-direction *P*_*y*_/*P*_*x*_ versus the stick number N (FWHM = 90°). The dashed vertical lines represent the optical transmission T = 99.2% where the electrical anisotropy quotient *ρ*_*a*_ exceeds the structural anisotropy quotient (*S*_*a*_ ≈ 2) and T = 97.5% where we observe electrical isotropy (*ρ*_*a*_ = 1) in Fig. 3b. (**b**) Simulated AgNW network representing a stick length of L = 19 *μ*m and an edge length of 40 *μ*m. Two different area coverages that correspond to T = 99.2 and 97.7% are shown. The highlighted paths express the pathway for the diffusion of the electrons that requires the lowest number of junctions (circles) with undesirable high junction resistance *R*_*j*_.

For further description of our experimental data, we refer on the computations with FWHM = 90° since the experimental angular distribution is in the same range (FWHM = 82°, Fig. 4a). In Fig. 6a we plot the ratio of the percolation probabilities *P*_*y*_/*P*_*x*_ obtained from Equation 5. Based on this plot we are able to explain the trend for *ρ*_*a*_(*T*) obtained in Fig. 3b. Hereby we distinguish between three sections (I-III) of *ρ*_*a*_(*T*) where two effects are differently pronounced. At very low area coverage (T > 99.2%), we have seen a strong increase of the electrical anisotropy *ρ*_*a*_ (Fig. 3b). Our computational approach results in significant decrease of *P*_*y*_/*P*_*x*_ for T > 99.2% (Fig. 6a). Hence, we attribute the drastic increase of the electrical anisotropy *ρ*_*a*_ for T > 99.2% to the lower percolation probability in y-direction than in x-direction (section I). For lower T, we obtain *P*_*y*_/*P*_*x*_ = 1. However, there still occurs decent electrical anisotropy between T = 99.2 and 97.5% as can be seen in Fig. 3b. Thus, there is another effect that influences *ρ*_*a*_. Structural anisotropy may not influence *P*_*y*_/*P*_*x*_ for T < 99.2%, but it still influences the pathways of the electrons along the network. Our analysis of the scanning force micrographs and Equation 4 results in a structural anisotropy quotient of *S*_*a*_ ≈ 2. Thus, the average orientation component *C*_*x*_ is twice *C*_*y*_. Figure 6b illustrates how this affects the network conductivity. Since the AgNWs exhibit dominant alignment in x-direction, less junctions are required for a continuous pathway in this direction than in case of a pathway in y-direction. As the intrinsic resistance *R*_*Ag*_ of the AgNWs is orders of magnitude lower than the contact resistance *R*_*j*_ of the junctions, a two-dimensional AgNW network can be regarded as an ensemble of contact resistances^14^. Therefore it is desirable that the electrons are allowed to move along the AgNWs without the need of passing a junction point, diffusing into another AgNW. Moderate alignment, which does not influence the percolation probability, increases the conductivity of the network as multiple works have shown^24^,^25^,^26^,^29^. However, due to the same effect, the conductivity vertical to the alignment (y-direction) is reduced. For low area coverage (T = 99.2%), significantly more junctions are required in y-direction than in x-direction (Fig. 6b). This effect dominates *ρ*_*a*_(*T*) between T = 99.2 and 97.5% (section II). It becomes less pronounced the more AgNWs are added to the network and at T = 97.5% we observe electrical isotropy (*ρ*_*a*_ ≈ 1, section III).

## Conclusion

This work represents the first experimental examination of electrical anisotropy within two-dimensional metallic nanowire networks close at the percolation threshold. Due to the excellent optoelectrical performance, AgNW networks are ideal paradigms for the description of stick percolation and the effects of stick alignment on electrical anisotropy. We found that liquid film coating such as dip-coating causes predominant alignment of the AgNWs in withdrawal direction. Structural anisotropy is not a significant drawback with respect to the technical usability in touch screens since our low-haze and ultra-transparent (T = 97–98%) AgNW films fulfill the industrial requirement of *R*_*s*_ < 100 Ω/sq in both directions.

The alignment of the AgNWs affects the electrical anisotropy only in case of very high optical transmission. We attribute this phenomenon to two different effects. The first one considers the number of junctions that are required for a continuous percolation path. Diffusion of the electrons along an AgNW is more desirable than diffusion into another AgNW. Due to the alignment of the AgNWs, less high-resistance junctions are required for diffusion in the withdrawal direction of the previous dip-coating process. The second and more pronounced effect on electrical anisotropy appears very close to the percolation threshold, where the percolation probability is higher with respect to parallel direction of the previous dip-coating process than orthogonal to it. The computational approach shows good agreement with the experimental data. This work shows that possible differences between the electrical conductivity parallel and orthogonal to the previous liquid film coating direction should be evaluated, when discussing films with low area coverage close at the percolation threshold.

## Experimental

Silver nanowires were purchased from Nanopyxis Ltd., South Korea. The as-delivered dispersion (1 w% Ag in ethanol, supplier information) has been diluted to 0.25 w%. Microspcopy slides (75 × 25 mm, Thermo Fischer) were used as substrates. The withdrawal velocity of the dip-coating process was 300 mm/min and one dip has been applied for each sample. The films were allowed to dry at room temperature. We produced 150 samples with different optical transmission. For each coating process two substrates were used and placed backside to backside at the mount of the dip-coater. Before each new pair of samples we have slightly diluted the dispersion with ethanol. Doing so, the final Ag concentration of the dispersion for the last samples was 0.06 w%. All films were annealed at 120° in an oven (Gestigkeit PR 5-3T) for 10 minutes.

Optical transmission has been measured at 550 nm (T80+, PG Instruments), with accuracy of Δ*T* = 0.1%. The optical transmission shown in this work exclude the optical transmission of the bare substrate. Two-probe and four-probe measurements appeared to coincide within the *R*_*s*_-range observed in this work, for which reason two-probe measurements have been applied. Scanning-force microscopy has been performed with a Veeco Dimension 3100 and antimony-doped silicon tips (TESPA-V2, Bruker Instruments). All scans were carried out in tapping mode (scan rate 0.3 Hz) and we show the height images. The orientation *θ* of the AgNWs has been analyzed with Gwyddion. The analysis of the experimental data has been carried out with IGOR Pro. As for the computational approach, the stick networks have been generated with Mathematica (see supporting information).

## Additional Information

**How to cite this article**: Ackermann, T. *et al*. The effect of rod orientation on electrical anisotropy in silver nanowire networks for ultra-transparent electrodes. *Sci. Rep.* **6**, 34289; doi: 10.1038/srep34289 (2016).

## Supplementary Material

Supplementary Information
