# Supplementary material for: The effect of rod orientation on electrical anisotropy in silver nanowire networks for ultra-transparent electrodes
Source: Sci Rep. 2016 Sep 28;6:34289. doi: 10.1038/srep34289 (PMC5039631; doi:10.1038/srep34289)
Supplement: Supplementary Information [file srep34289-s1.pdf]

The effect of rod orientation on electrical anisotropy in silver nanowire networks for ultra-transparent electrodes

Thomas Ackermann[1,2*], Raphael Neuhaus[2,3], and Siegmar Roth[4,5]

[1]Graduate School of Excellence in advanced Manufacturing Engineering, University of Stuttgart, Nobelstr.12, 70569 Stuttgart, Germany
[2]Fraunhofer Institute for Manufacturing Engineering and Automation, Nobelstr. 12, 70569 Stuttgart, Germany
[3]Institute for Industrial Manufacturing and Management, University of Stuttgart, Allmandring 35, 70569 Stuttgart, Germany
[4]Alan G. MacDiarmid NanoTech Institute, University of Texas at Dallas, 800 West Campbell Rd., Richardson, TX 75080, USA
[5]Sineurop Nanotech GmbH, Muenchner Freiheit 6, 80802 Munich, Germany
[*]thomas.ackermann@gsame.uni-stuttgart.de

SUPPORTING INFORMATION

Calculation of the Landau-Levich profile

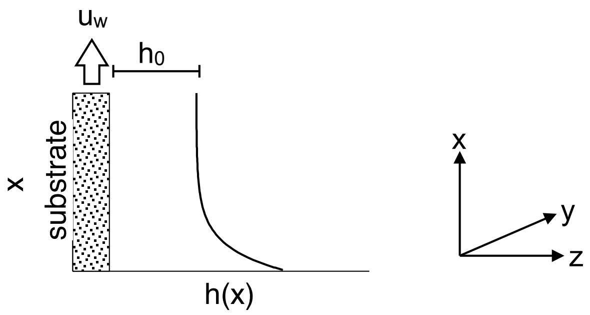

Figure S1. The Landau-Levich profile of the dip-coating process.

The shape of the meniscus within a dip-coating process, the so-called Landau-Levich profile depends on the the withdrawal velocity u_w and the fluid parameters viscosity η, surface tension γ and density ρ. The liquid film thickness h_0 depends on the Landau-Levich relation:

$$h_0 = 0.944 \cdot \frac{\eta^{2/3}}{\gamma^{1/6} (\rho g)^{1/2}} \cdot u^{2/3} = k_1 \cdot u^{2/3} \tag{1}$$

Probstein has deducted the Landau-Levich profile from fluid dynamics,[1] and the result is as follows.

$$h(x) = k_1 \cdot u_w^{2/3} \left[1 + exp \left(- \frac{x}{k_1 \cdot u_w^{2/3}} \left(\frac{3\eta u_w}{\gamma} \right)^{1/3} \right) \right] \tag{2}$$

Equation 2 has been used for the plot of the Landau-Levich profiles in Figure 1b of the paper.

[1]Probstein, R. F., Coating Flows, In: Probstein, G. F. (Ed.), *Physicochemical Hydrodynamics - An Introduction*, 10.3, 280-289, (Butterworth-Heinemann,1989).

Quality window for the transparent electrodes

The quality window for industrially acceptable transparent electrodes is often referred as optical transmission T > 90 % and electrical sheet resistance $R_s < 100\ \Omega$/sq.[2,3] However light scattering is a significant problem for the usability of AgNW films as transparent electrodes in displays since the films appear hazy. The extent of the light scattering and hence the haze depends on the area coverage and the diameter of the AgNWs. In Figure S2a we plot the optical haze H versus the optical transmission T. Haze was measured according to ASTM D1003, which defines Haze as the amount of the transmitted light that is scattered by an angle higher than 2.5 %. Acceptable haze that is not detectable to the human eye is represented by the dashed line. It is clearly seen that films made of thinner AgNWs exhibit lower haze at same T. Besides the high aspect ratio (L/d = 760), the low haze is a fundamental reason why we have used very thin AgNWs for the experiments shown in the paper (The AgNWs with d = 95 nm serve only as comparison within this supporting information and are not mentioned in the paper). As a result, the haze-corrected quality window is T > 97 % and $R_s < 100\ \Omega$/sq in case of AgNW films made of AgNWs with d = 25 nm. We consider such films as ”ultra-transparent”.

Figure S2b shows several transparent AgNW films. We note that the human eye detects haze better than the camera. However the first two films clearly appear hazy on the photographs, whereas the AgNW films used in the main paper (d (AgNW) = 25 nm, T > 97 %) exhibit significantly lower haze.

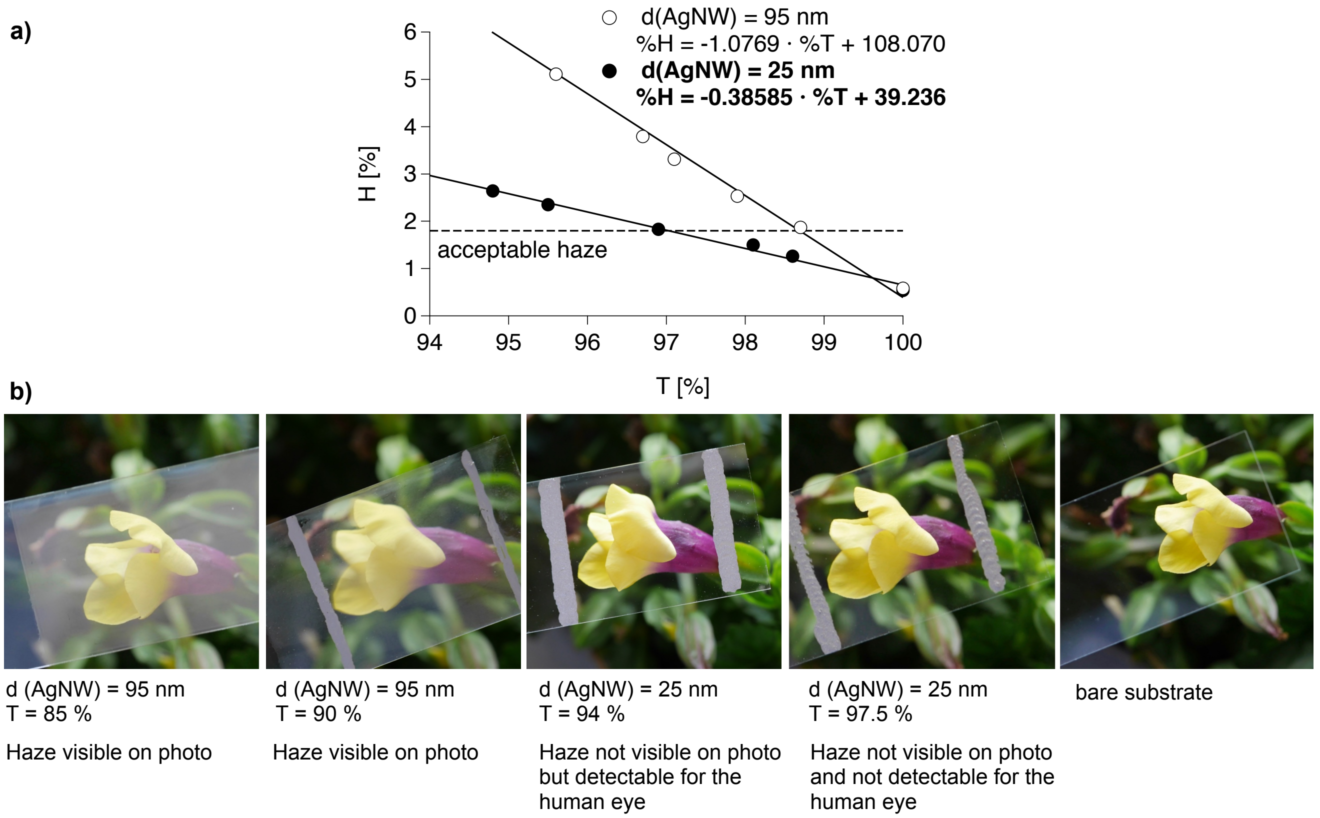

Figure S2. a) Optical haze H versus the optical transmission T. The haze of the bare substrate is H = 0.54 %. The data for T exclude the substrate. b) Several transparent AgNW films on glass substrates.

[2] De, S., King, P. J., Lyons, P. E., Khan, U. & Coleman, J. N. Size effects and the problem with percolation in nanostructured transparent conductors. *ACS Nano* **4**, 7064–7072 (2010).

[3] De, S. & Coleman, J. N. The effects of percolation in nanostructured transparent conductors. *MRS Bull.* **36**, 774–781 (2011).

Determination of the AgNW diameter

For the determination of the AgNW diameter we extracted profile lines from scanning-force micrographs (tapping mode, scan rate 0.63 Hz, 512 px) as shown in Figure S3 (white lines). The profiles are shown with the maxima of the peaks set to 0 in xy-direction. The z-directions of the surface next to the AgNWs were set to 0.

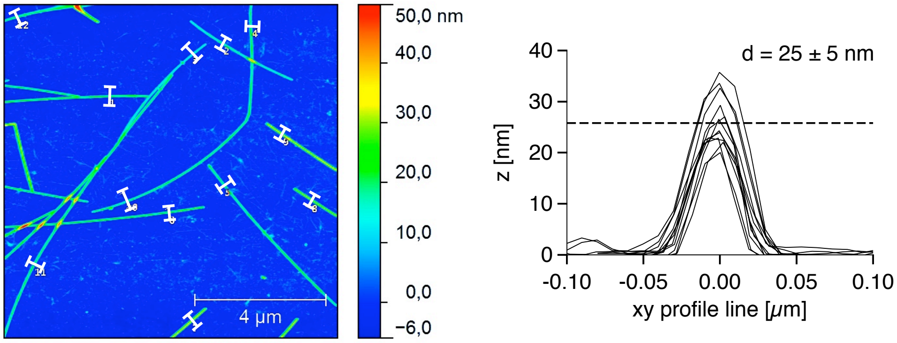

Figure S3. Scanning-force micrograph of an AgNW film at low area coverage and extracted profile lines.

Calculation of the area coverage from scanning force micrographs

The scanning force micrographs in the main paper appear to show a significant higher area coverage than we observe from the optical transmission T. In the following we will explain this phenomenon with artifacts in scanning force microscopy and compare the area coverage calculated from T to the area coverage of the scanning force micrograph.

In Figure S4a we show the same image as shown in the main paper. The optical transmission of this sample is T = 97.7 %. Following Equation 2 in the main paper, the area coverage is A = 2.6 %. The area coverage in Figure S4a appears much higher. In Figure S4b we plot the height (and color) distribution function ρ, which has been extracted using the image analysis software Gwyddion. We observe three sections of ρ: The height of the background (S_1), the height of the AgNWs (S_2) and the height of the AgNW junction points (S_3). The ostensive area coverage in Figure S4a is 39.3 % (S_2+S_3). This area coverage is about 15 times larger than the area coverage, which has been determined from transmission spectroscopy. In order to explain this huge difference, it is important to consider the effects, which influence the two methods. In transmission spectroscopy, the area coverage is calculated from T, including a correction parameter a_1 which considers light scattering (a_1 = 0.87 in case of d = 25 nm and wavelength λ = 550 nm).

$$\%A = 100 \cdot NLd = \frac{100 - \%T}{a_1} = \frac{100 - 97.7}{0.87} = 2.6 \tag{3}$$

One AgNW covers an area of Ld, where L is the length and d the diameter of the AgNW and A depends on the dimensions of the AgNWs and the number of AgNWs per area unit N. Theoretically, each junction of two crossing AgNWs reduces the covered area compared to the case where the AgNWs do not cross. In case of orthogonal crossing, the area of a junction is d^2 = $6.25 \cdot 10^{-5}$ μm^2. The covered area of the two AgNWs is 2Ld = 0.95 μm^2. It is obvious that the reduction of the area coverage caused by overlapping AgNWs at the junctions can be neglected. However, in scanning force microscopy the junctions cover 8.1 % of the area. This can be explained by tip artifacts. In Figure S4c we plot two enlarged sections of the image shown in Figure S4a and in Figure S4d we show the extracted height profiles. Especially section (1) exhibits several regions where junctions are shown much broader than they actually are. If junctions are closely neighbored, the probe tip will not detect the entire valley between these junctions as illustrated in the inset of section (1). As a result of this tip artifact, we observe height profiles which are not entirely separated from each other (arrows in Figure S4d). Since the junction points influence only the scanning force image but have only a neglectable influence on the real area coverage, we do not consider section S_3 in the height distribution function for the determination of A from the scanning force image and consider only section S_2, which is based on the height of the AgNWs without junctions. The area coverage of the AgNWs is 31.2 %. Hence, although ignoring tip artifacts caused by junctions, the area coverage A_{sfm} is still much larger than A = 2.6 % calculated from T. In the further we will explain this large difference.

One of the major advantages of scanning force microscopy is its very precise z-resolution, which allows us a very exact determination of the AgNW diameter d (Figure S3). However, it is also seen that the lateral width (xy plane) of the AgNWs is shown broader than 25 nm. This phenomenon is even more distinct in case of a larger image like Figure S4a. We have analyzed

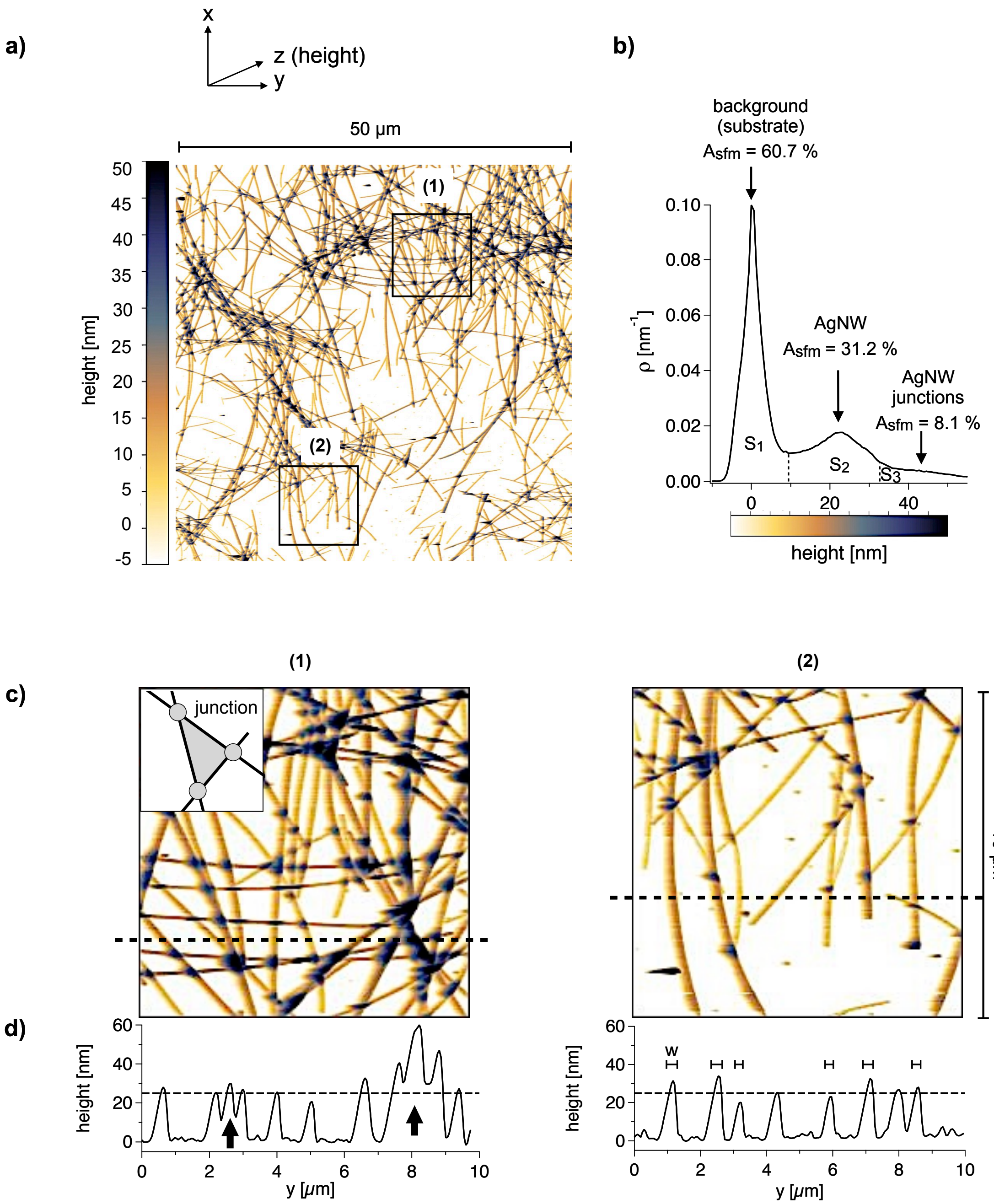

Figure S4. a) Scanning force micrograph (tapping mode, scan rate 0.3 Hz, 1024 px) of the sample with T = 97.7 % in Figure 4 of the main paper. b) The height distribution function ρ of a) where the maximum of the background distribution is at 0 nm. c) Enlarged sections from a). The dashed lines represent the location where height profiles are extracted. d) Extracted height profiles. The arrows in the height profile of (1) represent regions where the AgNWs are not entirely separated by the tip. The dashed line represents the average AgNW diameter d = 25 nm. The height profile of (2) also represents the width of those AgNWs which are oriented orthogonal to the scanning direction.

the width w of the AgNWs, which are oriented orthogonal to the scan direction (y) and found an average width of w = 286 nm (Figure S4d). The detected heights of the AgNWs show good agreement with d = 25 nm, which we have observed more precisely in Figure S3. We thus have to use a correction factor c_{sfm}

$$c_{sfm} = \frac{w}{d} = 11.4 \tag{4}$$

in order to compare the area coverage A_{sfm} found in scanning force microscopy with the area coverage observed in other measurements:

$$\%A_{sfm,corrected} = \frac{\%A_{sfm}}{c_{sfm}} = \frac{31.2}{11.4} = 2.7 \tag{5}$$

Considering artifacts in scanning force microscopy, the calculated area coverage in Equation 3 is almost similar to the area coverage determined from transmission spectroscopy (Equation 3). We have analyzed two other scanning force micrographs (Figure S5). After the consideration of the presented correction, the corrected area coverage from the scanning force micrographs exhibit also for these samples values which are comparable to the area coverage determined from transmission spectroscopy.

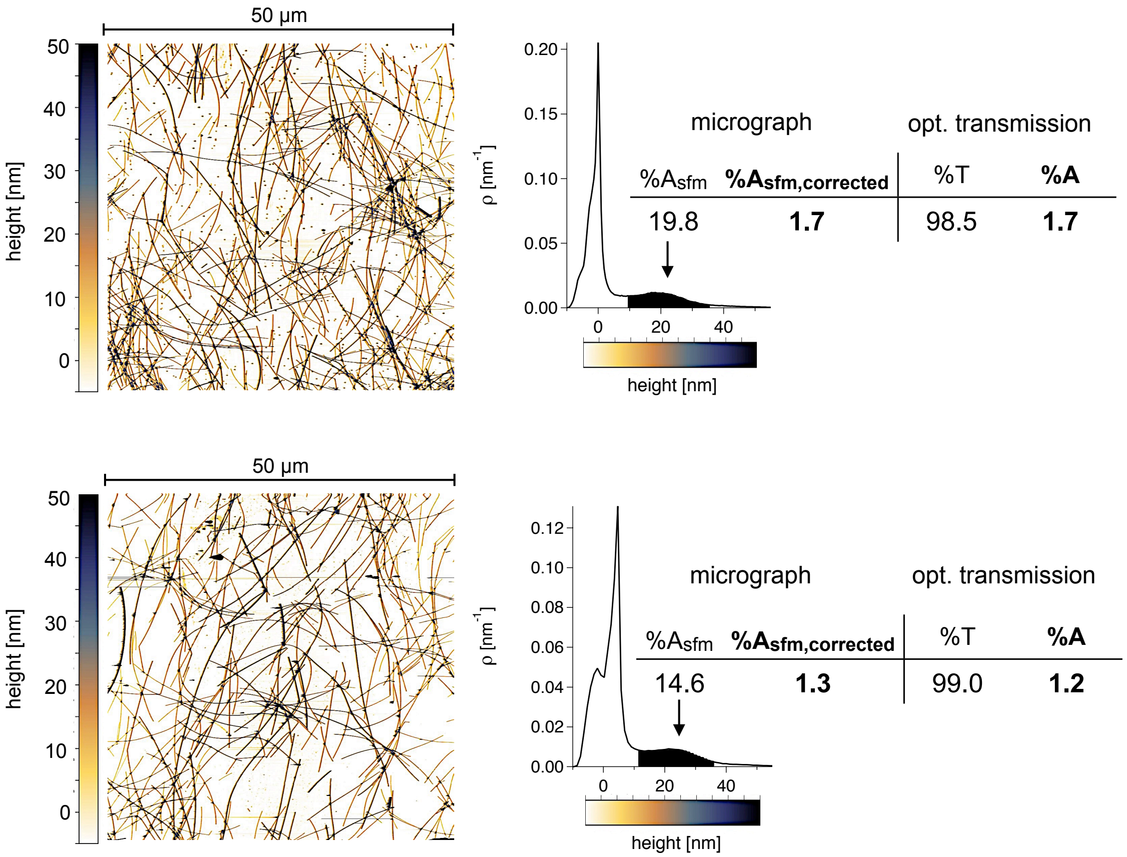

Figure S5. Scanning force micrographs (tapping mode, scan rate 0.3 Hz, 1024 px) of two further samples shown in Figure 4 of the main paper. The area coverage A_{sfm} is determined by integration of the height distribution function ρ and $A_{sfm,corrected}$ is calculated with Equation 5. The area coverage A is calculated from the optical transmission T with Equation 3.

Within the main paper all discussions are based on the area coverage determined from transmission spectroscopy due to the following two reasons: Firstly, we consider the area coverage determined from transmission spectroscopy as more reliable since this method observes a significantly larger surface area of the sample. Therefore we have a lower risk of detecting a spot with area coverage significantly bellow or above sample average. Secondly, the measurement of T is much easier and we have analyzed more samples (around 150) with transmission spectroscopy. Within the main paper, scanning force microscopy delivers information about the orientation angle of the AgNWs and about the AgNW diameter, rather than information about the area coverage. However, it is important to explain the huge difference between the area coverage found from T and the apparent area coverage of the AgNWs in the scanning force micrographs.

Orientation for higher area coverage

We have also determined the statistical orientation of the AgNWs in films with higher area coverage than in case of Figure 4a in the main paper. Due to the higher area coverage it is more difficult to analyse these samples. However, we have measured the orientation of 276 AgNWs. The FWHM is smaller than for lower area coverage (78 ° instead of 82 °) and the structural anisotropy quotient S_a is slightly higher (2.14 instead of 1.99). The insets of Figure S6 express how neighbored AgNWs (dashed) influence the alignment and how this effect becomes more distinct for higher area coverage.

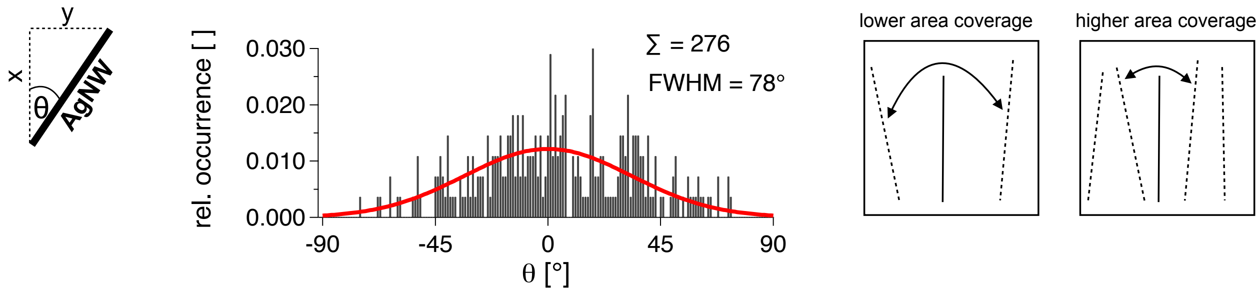

Figure S6. Distribution of the orientation angle θ for samples with higher area coverage. The optical transmission of the samples is between 98.1 and 99.0 %.

Computational approach with Mathematica®

```
In[378]:= SeedRandom[1]
          (*random number generation, [1] to [100]*)

          n = 140;
          (*number of points, respectively sticks*)

          centers = RandomReal[{0, 100}, {n, 2}];
          (*two dimensional seed of the randomly positioned
           points representing an area of 100x100 length units*)

          ListPlot[centers, PlotRange → {{0, 100}, {0, 100}},
           AspectRatio -> Automatic, Frame → True]
```

Out[381]=

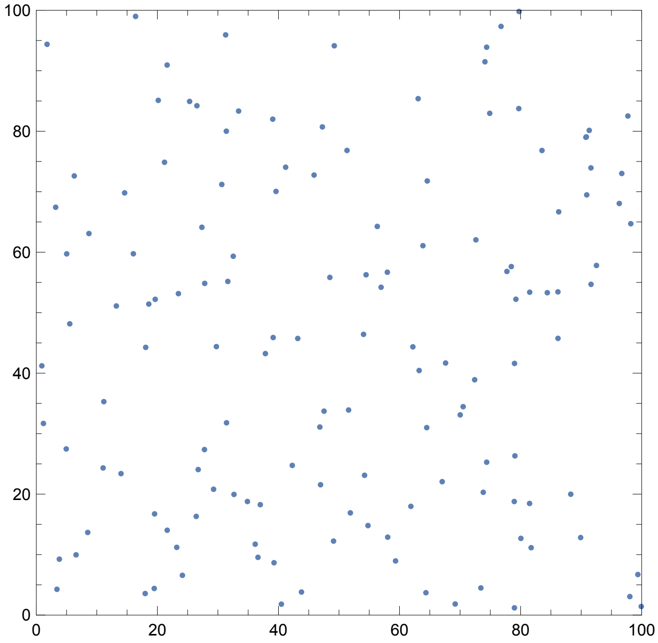

Figure S7. A random seed of n points is generated. In this example we seed 140 points and show the first of 100 simulations.

In[382]:=
```
θ = RandomVariate[NormalDistribution[π / 2, (1 / 2.3548) * π / 2], n];
(*NormalDistribution[μ,σ] with mean μ=
 π/2 and standard deviation σ =2*sqrt(2*ln2)*FWHM = 1/2.3548*FWHM*)
L = 19
(*length of the sticks, representing 19 length units*)

stickend1 = centers + Transpose[L {Cos[θ], Sin[θ]} / 2];
stickend2 = centers - Transpose[L {Cos[θ], Sin[θ]} / 2];

lines = Map[Line, Transpose[{stickend1, stickend2}]];
Graphics[{Thick, lines}, PlotRange → {{0, 100}, {0, 100}}, Frame → False]
```

Out[383]= 19

Out[387]=

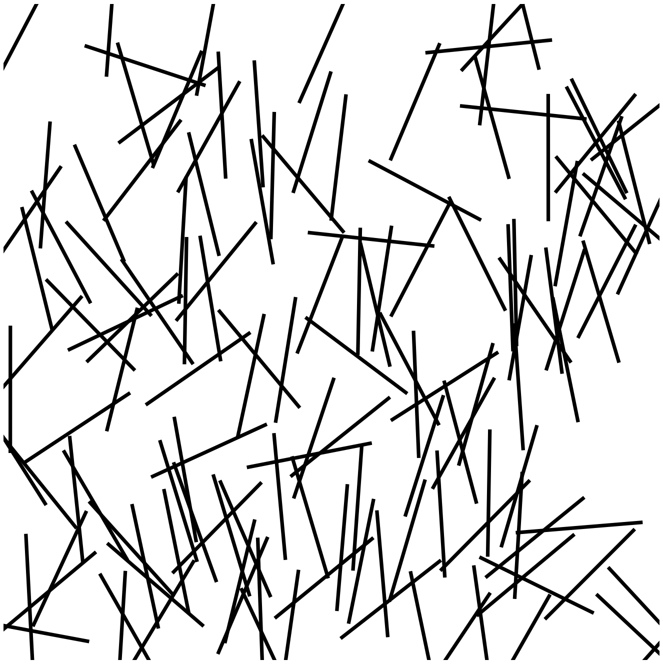

Figure S8. The angular distribution is defined as a normal distribution (Gaussian distribution): $G_\theta(\mu,\sigma) = \frac{1}{\sigma\sqrt{2\pi}} \cdot exp\left[\frac{(\mu-\theta)^2}{2\sigma^2}\right]$. In the paper we have set the mean of G_θ to $\mu = 0°$ in order to show the distribution between -90° and 90°, where perfect alignment of the AgNWs in withdrawal direction of the dip-coating process is $\theta = 0°$. For the same coordinates we have to use $\mu = 90°$ ($\pi/2$) in order to express the same since Mathematica® calculates the distribution from 0° to 180°. In case of Random distribution we applied θ = RandomReal[{-π/2,π/2},n];. Afterwards the sticks are generated by defining stickends. The connection between the stickends is the sticklength L, which is set as L = 19 unit lengths. In this example the FWHM of the angular distribution is 90° ($\pi/2$ in radians).

In[388]:=
```
lineGraphics = Graphics[lines, PlotRange → {{0, 100}, {0, 100}}, Frame → False];
ColorNegate@Colorize[MorphologicalComponents[ColorNegate@lineGraphics]]
```

Out[388]=

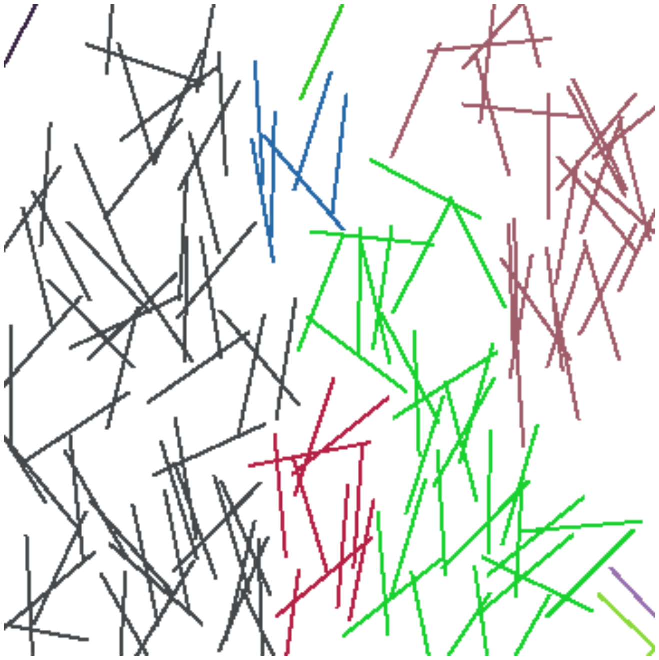

Figure S9. We processed the image applying mathematical morphology. That way, continuous paths are shown in different colors.
